# Women improving nutrition through self-help groups in India: Does nutrition information help?

**DOI:** 10.1016/j.foodpol.2024.102716

**Published:** 2024-10

**Authors:** Neha Kumar, Kalyani Raghunathan, Agnes Quisumbing, Samuel Scott, Purnima Menon, Giang Thai, Shivani Gupta, Carly Nichols, the WINGS study team

**Affiliations:** a Food and Nutrition Policy Department, International Food Policy Research Institute, Washington, DC, USA; b Food and Nutrition Policy Department, International Food Policy Research Institute, New Delhi, India; c Department of Applied Economics, University of Minnesota, Twin Cities, USA; d Department of Agricultural and Applied Economics, University of Georgia, Athens, USA; e Department of Geography, National University of Singapore, Singapore

**Keywords:** Maternal nutrition, Nutrition sensitive agriculture, Women’s self-help groups, India

## Abstract

Women’s self-help groups (SHGs) are an important platform for reaching poor women in India. Despite SHGs' women-focused programming, evidence of the impact of SHG-based interventions on nutrition outcomes is limited, and most evaluations of nutrition interventions have not examined intermediate outcomes along the impact pathways or outcomes for women themselves. This paper evaluates the effectiveness of an integrated agriculture-nutrition intervention delivered through women’s SHGs in five states in central and eastern India. The interventions involved the delivery of nutrition behavior change communication to groups through participatory approaches, community engagement around key issues, and the strengthening of collective organizations. Our analysis is based on three rounds of rich panel data on close to 2700 rural women and their households from eight districts in these five states and qualitative work from an accompanying process evaluation. Using difference-in-difference models with nearest neighbor matching methods, we present results on women’s anthropometry and diet-related outcomes.

We do not observe any improvements in women’s BMI or overall dietary diversity. Although more women in the nutrition intensification arm consumed animal source foods, nuts and seeds, and fruits, this was not enough to increase overall dietary diversity scores or the proportion of women achieving minimum dietary diversity. We measure intermediate outcomes along the program’s impact pathways and find improvements in household incomes, cultivation of home gardens, and utilization of government schemes but not in women’s empowerment. The lack of improvement in anthropometry and diets despite changes in some intermediate outcomes can be attributed to several factors such as low implementation intensity, poor facilitator capacity and incentives, the lack of relevance of the BCC topics to the average SHG member, and resource and agency constraints to adoption of recommended practices. Although we do not have data to test the parallel trends assumption and so do not interpret our results as causal, these findings do suggest that optimism about using group-based platforms needs to be tempered in resource-poor contexts.

## Highlights

• India continues to contribute greatly to the global prevalence of undernutrition.
• Women’s self-help groups (SHGs)- an important platform for reaching women in India.
• Evidence on effectiveness of SHG-based interventions on nutrition remains limited.
• Effectiveness of an agriculture-nutrition intervention delivered by women’s SHGs.
• Analysis based on three rounds of panel data on about 2500 rural women.
• No impact on women’s BMI or diet diversity, but they consumed more nutritious foods.
• Find improvements along several impact pathways – income, agriculture and rights.

## 1 Introduction

More than half the undernourished people in the world live in Asia (FAO, 2021), the bulk of those in India. Data from India’s fifth National Family Health Survey (NFHS-5) shows limited progress in key maternal and child health and nutrition indicators over the five years between 2015–16 and 2019–21 (IIPS & ICF 2017 & 2021). Whether India’s nutrition and social protection programs can buffer against slowing economic growth, increasing inequalities (Chatterjee, 2021), and the persistent effects of the COVID-19 pandemic (Headey et al., 2020) is a major concern, particularly for women and children who bear the disproportionate burden both of malnutrition and of the recent pandemic (IIPS & ICF, 2021; Bertrand et al., 2020; Deshpande, 2020, 2022).

Against this backdrop, there is great interest in using existing platforms that may be well-suited to delivering nutrition interventions at scale to vulnerable groups. One such platform is women’s groups, increasingly used to deliver development interventions both in South Asia and globally. Self-help groups (SHGs), the most prevalent type of women’s groups in India, are collectives of 10–20 women who meet regularly to save small amounts of money in a common account used to make microloans to group members. As SHGs mature, they are provided additional lines of credit from supporting NGOs or formal credit institutions. While the core function of these groups is savings and credit, they routinely engage in interventions around agriculture and livelihoods. Many supporting organizations now use group-based platforms to deliver other interventions—including nutrition interventions—to improve women’s livelihoods and well-being and to enhance women’s empowerment and gender equality (Desai et al., 2020; Diaz-Martin et al., 2023).

Our paper evaluates the effectiveness of integrated agriculture-nutrition interventions delivered through women’s SHGs in five states in central and eastern India. The interventions involved the delivery of nutrition behavior change communication (BCC) to groups through participatory approaches, community engagement around key issues, and the strengthening of collective organizations (SHGs and their higher order federations at the village and block levels). We collect three rounds of survey data on close to 2700 rural women and their households from eight districts in these five states. Using difference-in-difference models with nearest neighbor matching methods, we measure improvements in women’s anthropometry and diet-related outcomes. We then use our rich survey data and qualitative work from an associated process evaluation to measure intermediate outcomes along the program’s impact pathways to better understand the findings.

Evidence on the use of women’s groups to improve nutrition outcomes in India is small but steadily growing (see Desai et al., 2020, Raghunathan and Desai, 2021 for reviews). Interventions are of two types: those that layer nutrition interventions onto existing savings and credit groups like SHGs, and those aimed at community mobilization. Evidence on the impact of these approaches is mixed, and the bulk of it comes from evaluations of the JEEViKA program in Bihar or the Uttar Pradesh Community Mobilization Program. Programs that provided nutrition behavior change communication (BCC) − with or without individual counselling or supply-side interventions – noted impacts on knowledge, self-reported dietary diversity, infant and young child feeding practices (IYCF), and service utilization (Darmstadt et al., 2020, Hazra et al., 2020, Irani et al., 2016, Irani et al., 2021, Mehta et al., 2020b, Mehta et al., 2020a, Nair et al., 2017, Raghunathan et al., 2023, Saha et al., 2013). However, evidence of impact on women or child anthropometric outcomes is limited. Gope et al. (2019), a notable exception, found a 27% reduction in child wasting from an intervention that combined nutrition BCC through participatory learning and action cycles with other individually targeted interventions like home visits and creches for children. High and sustained intensity of implementation is key to achieving impact in thematic areas that are not core to group functioning (Hazra et al., 2022), but sustaining this intensity is difficult if other interventions are delivered through the same platform (Irani et al., 2021) or if efforts are scaled up too quickly (Majumdar et al., 2017).

A companion analysis to this study that uses cross-sectional data on a sample of mothers with young children complements this work in finding improvements in knowledge around maternal nutrition and IYCF, though these did not translate into improvements in practices or children’s anthropometry (Scott et al., 2022). Gains along knowledge-related dimensions, while beneficial, may have been insufficient to change behaviors, especially without any in-kind or cash transfers.

Finally, while the benefits of nutrition-sensitive agriculture programs for child and household diet diversity and increased consumption of specific target foods are well known (Ruel et al., 2018, Sharma et al., 2021), evidence of their impact on women’s nutrition and diets remains weak, partly because few studies examine *women’s* anthropometry or diet. This reflects the traditional focus on women as caregivers—with an instrumental role in improving nutrition—rather than a concern about women themselves.

Our paper contributes to the discussion of the feasibility of leveraging women’s SHGs (hereafter just SHGs)^2^ for nutrition-sensitive agriculture interventions that both improve rural livelihoods and reduce malnutrition of women and children. Interventions delivered through existing SHGs that were already engaged in extension and other activities related to agriculture and livelihoods are important for several reasons. First, these groups deliberately target and engage women, often from poor and marginalized groups, with implications for women’s empowerment, their own nutritional outcomes, and the nutritional outcomes of their children (Santoso et al., 2019). Second, the high rate of coverage of SHGs facilitates scalability and cost-effectiveness (Khanna and Majumdar, 2018). In India, SHGs supported by the National Rural Livelihoods Mission (NRLM) reach an estimated 84.5 million households (approximately 400 million individuals) at present^3^; still more women are reached by NGO-led groups. Third, these interventions build upon groups’ existing engagement around agriculture and associated livelihoods, which is important for the income and livelihoods of a sizeable portion of India’s population, including women, making it a strategic entry point for nutrition interventions (Ruel and Alderman, 2013). Fourth, they can potentially trigger multiple pathways to improve nutrition (Kumar et al., 2018). These include increasing incomes through savings and credit activities, increasing agricultural productivity through extension and other activities, delivery of nutrition BCC to directly influence individual and household behaviors, increased use of existing government services through improved awareness of rights and entitlements, and a cross-cutting pathway of improved women’s empowerment and collective action.

## 2 Conceptual framework and intervention description

### 2.1 Conceptual framework

**Fig. 1:**
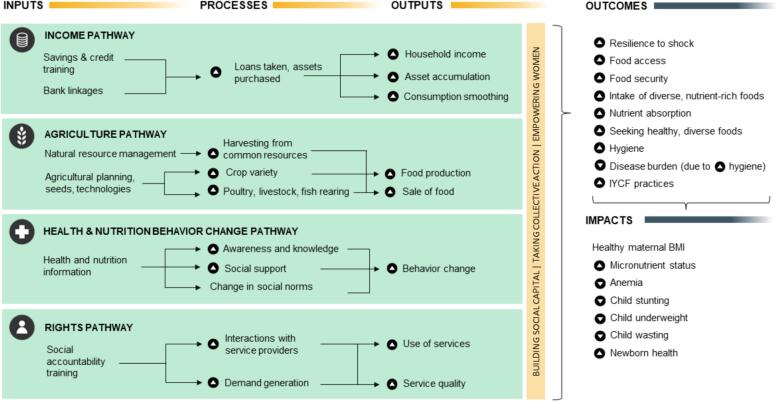
Theory of change and hypothesized pathways to impact for women’s group interventions.

The conceptual framework in (Kumar et al., 2018) lays out pathways from women’s group-based interventions to improved nutrition outcomes, working through income, agriculture, health and nutrition behavior change, and rights and entitlements (Fig. 1). The framework also includes a cross-cutting pathway revolving around building social capital, taking action collectively, and empowering women. In theory, a group-based intervention could trigger any or all these pathways depending on the programmatic inputs provided. Our previous work has explored different potential pathways to impact, e.g., the income pathway (Raghunathan et al., 2022), the agriculture pathway (Raghunathan et al., 2019, the rights pathway (Kumar et al., 2019) as well as cross-cutting pathways working through women’s empowerment (Kumar et al., 2021).

Source:

### 2.2 Intervention description

We evaluate a nutrition-sensitive agriculture intervention designed and implemented by Professional Assistance for Development Action (PRADAN) and the Public Health Resource Society (PHRS). PRADAN has supported the formation and strengthening of women’s savings and credit SHGs in multiple states of India since the early 1980s. As these SHGs mature, PRADAN links them to the formal financial sector, allowing them to obtain lines of credit from banks and from the government through NRLM and other development programs. Once the savings and credit activities are regularized, SHGs participate in agriculture and related livelihoods activities, such as agricultural extension and the adoption of technologies to enhance income generation, the use and purchase of modern inputs and machinery, creation of irrigation infrastructure and land reclamation, among others. PRADAN’s governance and rights activities include improving women’s political participation and strengthening grassroots democracy, stakeholder sensitization to the needs of poor rural women, supporting women’s land rights, and enhancing awareness and use of government entitlement schemes. Finally, PRADAN’s gender-oriented activities focus on promoting equality within the household, reducing violence against women, and supporting the role of women in income generating activities. While PRADAN does not target or specifically engage men within communities, PRADAN’s approach encourages SHG members to question discriminatory ideas and practices and build individual, household, and community-level awareness of gender issues, thereby addressing deep-rooted patriarchal norms and customs. SHGs are federated into Village Organizations, with representation from all SHGs within a village, and then to Gram Panchayat and Block-level Federations. Higher federations of SHGs undertake community mobilization and development activities, organizing large-scale events for their members and facilitating collective action around common goals.

Recognizing that the core set of livelihoods-focused interventions may not translate into improvements in health and nutrition for women within rural households, PRADAN partnered with PHRS in 2014–15 to design a set of health- and nutrition-focused BCC interventions that could be delivered using the existing agriculture-focused women’s SHGs. Starting in 2016, this nutrition-intensification (NI) model was layered onto the standard PRADAN activities in select intervention areas and included both nutrition-specific and nutrition-sensitive messaging delivered by trained local volunteers at monthly SHG meetings using an interactive participatory approach. SHG members were envisaged as agents of change who would share this information within their communities, making the intervention cost-effective, scalable, and sustainable. PHRS and PRADAN also strengthened the capacity of the Village Organization and other federations to engage the broader community on health and nutrition issues. These federations represented the concerns of SHG women to government officials and helped coordinate community-based events such as body mass index (BMI) camps, handwashing camps, and visits to local markets and ration shops.

The NI model interventions had components both added onto and integrated into the standard PRADAN interventions. The main additional component was the provision of health and nutrition BCC messages at monthly SHG meetings through a dedicated volunteer cadre of women known as Poshan Sakhis (henceforth PS), literally “nutrition friend”. To facilitate information dissemination and behavior change, PHRS trained and embedded one Block Program Officer in each PRADAN block team whose role was to anchor the nutrition work in that area. Each Block Program Officer trained and supported 5–8 ‘Mentors’, paid staff who oversaw the work of between 5 and 25 PSs each. The number of PSs ranged from 1 to 15 per village, depending on village size and spread and the number of SHGs. The BCC emphasized three key topics: the causes, consequences, and opportunities to prevent malnutrition; recommended IYCF practices, and appropriate diets of household members, especially women, and used innovative and participatory pedagogical techniques such as embedding messages within stories involving key recurring characters, group exercises, and meetings dedicated to community-led discussions of action points and next steps. Issues included in the curriculum built on community needs assessments and considered the expressed interests and needs of the partnering organizations. The approach used in the NI intervention followed Participatory Learning and Action (PLA) approaches, except that they were implemented within the women’s group rather than with the entire community. After training on the BCC content by the PRADAN team and PHRS staff, PSs disseminated this content to the SHGs under their purview. The Mentors monitored PSs’ performance and provided additional assistance and advice as needed. PSs also disseminated information about the community health and nutrition events organized by the higher-level federations in the SHG meetings.

NI interventions were also integrated into pre-existing standard program activities. For example, agriculture and livelihoods-related discussions focused not just on improving production, income, and food security, as in the PRADAN Standard model, but also incorporated information on production practices that could improve diets, such as enhanced production diversity, the promotion of non-cereal crops, especially pulses, and the importance of homestead kitchen gardens to enhance dietary quality. Governance and entitlements interventions were expanded to include awareness and utilization of those programs specifically targeted towards improving household food security, or towards the well-being of pregnant and lactating mothers, such as pregnancy registration and antenatal care services. Finally, gender-related discussions were broadened to include women’s roles in decision-making around agriculture, nutrition, and childcare, and equitable distribution of food within the household, among other topics.

## 3 Data and methods

### 3.1 Data

**Fig. 2:**
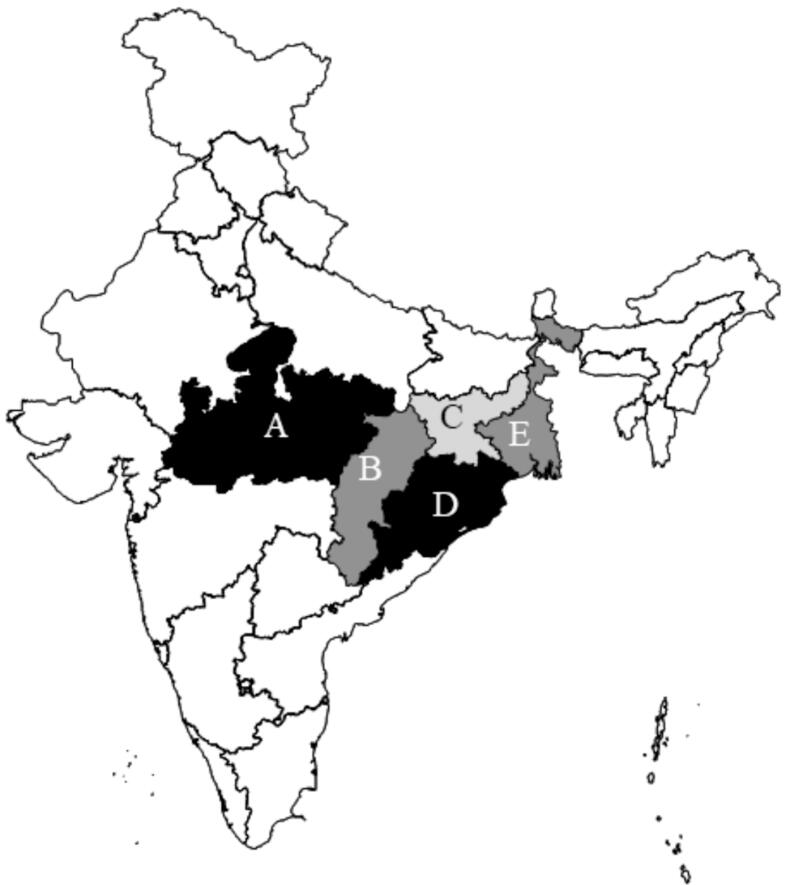
Map of India, shaded states indicate the study areas. Notes: 1. The boundaries and names shown, and the designations used on this map do not imply official endorsement or acceptance by the International Food Policy Research Institute (IFPRI). 2. A: Madhya Pradesh (2 districts), B: Chhattisgarh (1 district), C: Jharkhand (2 districts), D: Odisha (2 districts), E: West Bengal (1 district). See text for more details.

Our study was conducted in eight districts of five central and eastern Indian states: Dindori, Mandla (Madhya Pradesh), Bastar (Chhattisgarh), Purulia (West Bengal), Kandhamal, Rayagada (Odisha), West Singbhum, Dumka (Jharkhand) (Fig. 2) and employed a multi-stage sampling strategy to select target households. In the first stage, three blocks were selected from each district – one block where PRADAN conducted its standard activities, the PRADAN Standard arm; one where the nutrition interventions were layered on top of the standard activities, the NI arm; and a comparison block without PRADAN presence, the Comparison arm. SHGs in the Comparison arm were supported by other governmental or non-governmental organizations.

Consistent with PRADAN’s ‘bottoms-up’ approach to programming, the NI model was not randomly assigned but was instead implemented in areas where PRADAN teams expressed an active interest in addressing undernutrition. The nonrandom program placement raises concerns of bias because the program may be placed in more “receptive” communities. To address this, our evaluation design matched the standard PRADAN and Comparison blocks to the pre-determined NI block using a set of demographic, economic, infrastructure, standard of living, and agriculture indices drawn from secondary data and compiled in Indicus Analytics (2013). See (Raghunathan et al., 2022) for more details on this process. This matching exercise resulted in three matched blocks per district, one from each arm, for a total of 24 blocks.

In the second stage, in each of the two PRADAN arms, Standard and NI, we chose five villages at random from the full list of villages where PRADAN was operational. In the Comparison arm, we chose seven villages at random from the full list of villages. We oversampled Comparison arm villages to improve the matching on observables (see section 3.2). In the 136 villages thus selected to be in the study, we conducted a full listing of households to identify those satisfying the study eligibility criterion of having at least one ever-married female member between 15 and 49 years old. In the final stage of sampling, 20 households were chosen at random from the list of eligible households in each village. This design resulted in a total planned sample of 2720 households, 800 in each of the two treatment arms and 1120 in the Comparison arm.

We also conducted a mixed methods process evaluation as part of the study. The quantitative portion used templates to collect data on implementation at the block level while the qualitative study (Nichols, 2021a, Nichols, 2021b) was conceptualized to understand findings from the midline survey (November 2017-January 2018). The qualitative study focused on two of eight PRADAN NI sites, Bastar District, Chhattisgarh and Purulia District, West Bengal. These two blocks were chosen for maximum variability based on program evaluation data and characteristics of PRADAN’s engagement to understand enabling factors and barriers of the same program in different environmental and socioeconomic contexts (Nichols, 2021a). The same protocols for sampling and data collection were used in both sites. Data was collected through semi-structured interviews and focus groups from four distinct groups: PRADAN professionals, mentors, nutrition volunteers, and SHG members, using the same strategy across sites. Interviews were transcribed, translated, and uploaded to MAXQDA 2018 for analysis using inductive coding. See (Nichols, 2021a, Nichols, 2021b) for further details.

This paper uses two rounds of panel household data from baseline and endline surveys. To maintain seasonal comparability, the two rounds were conducted at the same time of year; the baseline between mid-November 2015 and end-January 2016 (prior to intervention rollout in 2016) and the endline between early-December 2019 and early-February 2020. In both rounds, individual- and household-level questionnaires were administered to the main female respondent and to an adult male (age>15 years), preferably the spouse of the respondent woman. We also administered a community (village-level) questionnaire to collect data on community characteristics at both baseline and endline.

The final achieved baseline sample included 2744 households. Not all women in our sample were SHG members at baseline, since membership was not an explicit eligibility criterion, and women could join (or leave) SHGs between baseline and endline. At baseline, 45% of the women sampled in the NI arm belonged to SHGs, compared to only 34% in the Standard arm, and 39% in the Comparison arm (Table 2). These differences across arms were statistically significant. Despite this, our sampling approach achieved balance across individual and household characteristics at baseline, with a few exceptions, mitigating concerns of bias arising from PRADAN’s non-random program placement.

The study received ethical approval from the Institutional Review Board of the International Food Policy Research Institute, (IRB approval number 00007490) and was registered on the Registry for International Development Evaluations (RIDIE-STUDY-ID-5d567e7e8b96).

### 3.2 Attrition

At endline, we reinterviewed 2489 of the 2744 baseline households for an overall attrition rate of approximately 9 percent. We test for selective attrition for the respondent woman sample; characteristics that differ between the attrited and non-attrited households include respondent women’s marital status, presence of mother-in-law, SHG membership and bank account ownership. However, these are not correlated with treatment assignment, and thus are unlikely to bias the impact estimates. Additional data were collected to compute the Abbreviated Women’s Empowerment in Agriculture Index (A-WEAI) (Alkire et al., 2013; Malapit et al., 2017) for a subsample of baseline respondents for whom a male respondent was available for interview. This subsample was substantially different from the overall sample, necessitating the use of sample selection weights.

### 3.3 Empirical strategy

Our study specifically aims to assess: (1) the overall impact of PRADAN’s Standard and NI intervention models on women’s dietary diversity and BMI, relative to the Comparison arm; (2) the *additional impact* of the NI model over and above the Standard model on women’s dietary diversity and BMI; and (3) PRADAN’s overall impact and the additional NI impact on factors along the hypothesized pathways to impact − nutrition knowledge, agriculture, income, rights, empowerment.

We use block level matching to account for potential bias in PRADAN’s program placement. Despite overall balance achieved by this approach, bias may still arise from differences in unobserved characteristics across arms. We therefore combine block-level matching with nearest neighbor matching at the individual level to construct an appropriate comparison group for each research question.

Nearest neighbor matching is a form of covariate matching in which the comparison group of nonbeneficiaries is selected based on similarity to the beneficiary households on observable characteristics (Abadie et al., 2004, Abadie and Imbens, 2006). We use a set of observable baseline household and village characteristics (presented in Table 1, excluding the SHG membership indicator) to match each household in a treatment block to five households in the comparison block based on their degree of similarity, determined using a weighted function of the covariates for each observation. Since we use more than two continuous covariates, we apply the bias correction method proposed by Abadie and Imbens (2006). Where both baseline and endline data are available, we estimate the difference-in-difference estimates of impacts by comparing changes in outcomes over time between treatment and comparison arms. When only endline values of an indicator were collected, we use a single difference model to estimate impacts.

**Table 1:** Household characteristics at baseline by treatment arm.

	**Full sample baseline means**		
	**NI**	**Standard**	**Comparison**
**Respondent woman characteristics**			
Age, years	33.24	32.66	33.10
	(8.17)	(8.54)	(8.25)
Age squared	1,171.76	1,139.36	1,163.31
	(556.82)	(582.87)	(559.46)
Years of schooling	2.16	2.30	2.30
	(3.45)	(3.47)	(3.69)
Years of schooling squared	16.58	17.30	18.89
	(32.17)	(31.73)	(37.50)
Is employed	0.75^a^	0.69^a|c^	0.76^c^
	(0.44)	(0.46)	(0.43)
Married	0.92	0.94	0.93
	(0.27)	(0.24)	(0.25)
Number of years married	14.42	13.89	14.24
	(9.46)	(9.59)	(9.46)
Mother-in-law present	0.21	0.21	0.21
	(0.41)	(0.41)	(0.41)
Father-in-law present	0.11^b^	0.12^c^	0.15^b|c^
	(0.32)	(0.32)	(0.36)
Currently belong to a SHG	0.45^a|b^	0.34^a|c^	0.39^b|c^
	(0.50)	(0.47)	(0.49)
**Household characteristics**			
Household head is Scheduled Tribe or Scheduled Caste	0.75^b^	0.77^c^	0.83^b|c^
	(0.44)	(0.42)	(0.38)
Household head is OBC	0.18^b^	0.20^c^	0.14^b|c^
	(0.39)	(0.40)	(0.34)
No. female members	2.36	2.34^c^	2.44^c^
	(1.21)	(1.18)	(1.26)
No. male members	2.22	2.25	2.29
	(1.15)	(1.15)	(1.17)
Ratio of < 15yrs and > 55yrs to 16-55yrs	1.48	1.38	1.40
	(1.37)	(1.22)	(1.30)
HH dwelling score	1.09^b^	1.10^c^	0.98^b|c^
	(0.87)	(0.80)	(0.79)
**Village characteristics**			
Average years of education among women	2.20	2.38	2.30
	(1.05)	(1.20)	(1.59)
Village has at least one government primary school	0.90	0.90	0.89
	(0.31)	(0.30)	(0.32)
Village has at least one private primary school	0.10	0.05	0.06
	(0.31)	(0.22)	(0.23)
Village has at least one Anganwadi center	0.90	0.90	0.89
	(0.31)	(0.30)	(0.32)
Electricity in all areas	0.79	0.76	0.83
	(0.41)	(0.43)	(0.38)
Distance to nearest town < 20 km	0.54	0.67	0.54
	(0.51)	(0.48)	(0.50)
*N*	733	771	985

Source: Authors' calculations. Notes: Standard deviation in parentheses. Superscripts a, b and c denote significant differences (p < 0.1) between NI and Standard arms, NI and Comparison arms, and Standard and Comparison arms, respectively. The household dwelling score (0–3) is a simple row total of binary indicators on the household having an improved roof, floor, and walls. An Anganwadi center is a rural childcare center under the national Integrated Child Development Services program, where pregnant and lactating mothers, children, and adolescents receive a variety of health, nutrition, and immunization services.

**Table 2:** Summary statistics and DID estimates for key respondent woman outcomes, by treatment arm.

	**Baseline Means**			**Endline Means**			**DID**	
	**NI**	**Standard**	**Comparison**	**NI**	**Standard**	**Comparison**	**PRADAN vs Comparison**	**NI vs Standard**
	(1)	(2)	(3)	(4)	(5)	(6)	(7)	(8)
**PANEL A: Anthropometry**								
Body mass index (BMI)	19.23^b^	19.28^c^	18.92^b|c^	19.63	19.79^c^	19.48^c^	−0.026	−0.061
	(2.77)	(2.70)	(2.41)	(3.13)	(3.03)	(2.68)	(0.077)	(0.102)
Whether underweight	0.46	0.45	0.48	0.41	0.37	0.39	0.017	0.034
	(0.50)	(0.50)	(0.50)	(0.49)	(0.48)	(0.49)	(0.022)	(0.030)
Whether of average weight	0.50	0.51	0.50	0.53^b^	0.56	0.58^b^	−0.03	−0.03
	(0.50)	(0.50)	(0.50)	(0.50)	(0.50)	(0.49)	(0.02)	(0.03)
Whether overweight	0.04^b^	0.04^c^	0.02^b|c^	0.06^b^	0.07^c^	0.03^b|c^	0.01	−0.01
	(0.21)	(0.19)	(0.15)	(0.24)	(0.25)	(0.18)	(0.01)	(0.01)
**PANEL B: Dietary diversity**								
Total number of food groups consumed in last 24 h	2.84^a^	2.98^a|c^	2.83^c^	3.35	3.37^c^	3.26^c^	0.031	0.124
	(1.21)	(1.28)	(1.18)	(1.10)	(1.00)	(1.05)	(0.069)	(0.089)
Achieved minimum dietary diversity (>=5 food groups)	0.09^a^	0.13^a|c^	0.08^c^	0.13	0.13	0.12	−0.005	0.015
	(0.29)	(0.33)	(0.27)	(0.34)	(0.33)	(0.32)	(0.020)	(0.026)
Ate any animal source food in last 24 h	0.17^a^	0.23^a|c^	0.18^c^	0.25^b^	0.23	0.21^b^	−0.019	0.059*
	(0.38)	(0.42)	(0.39)	(0.43)	(0.42)	(0.41)	(0.025)	(0.031)
**PANEL C: Diet composition**								
Starchy staple foods	0.99	0.99	0.99	1.00	1.00	1.00	0.009*	0.004
	(0.08)	(0.11)	(0.10)	(0.04)	(0.05)	(0.06)	(0.005)	(0.008)
Pulses	0.51^b^	0.50^c^	0.45^b|c^	0.64^a|b^	0.71^a^	0.69^b^	−0.077**	−0.047
	(0.50)	(0.50)	(0.50)	(0.48)	(0.45)	(0.46)	(0.032)	(0.040)
Nuts and seeds	0.01	0.02	0.02	0.03^a^	0.02^a^	0.02	0.021**	0.024**
	(0.10)	(0.12)	(0.13)	(0.18)	(0.12)	(0.15)	(0.010)	(0.010)
Dairy products	0.08^a^	0.10^a^	0.10	0.08	0.08	0.09	−0.005	0.009
	(0.27)	(0.31)	(0.30)	(0.27)	(0.28)	(0.29)	(0.015)	(0.020)
Flesh foods	0.07^a^	0.12^a|c^	0.07^c^	0.15^b^	0.14	0.11^b^	−0.030	0.070***
	(0.26)	(0.32)	(0.26)	(0.36)	(0.34)	(0.32)	(0.020)	(0.026)
Eggs	0.03	0.03	0.03	0.04	0.04	0.03	0.014	−0.006
	(0.16)	(0.17)	(0.16)	(0.19)	(0.19)	(0.16)	(0.011)	(0.012)
Dark green leafy vegetables	0.45	0.48	0.48	0.35	0.36	0.35	0.041	0.009
	(0.50)	(0.50)	(0.50)	(0.48)	(0.48)	(0.48)	(0.030)	(0.040)
Other vitamin A-rich vegetables and fruits	0.18	0.17	0.15	0.14^b^	0.14^c^	0.09^b|c^	0.035	−0.024
	(0.38)	(0.37)	(0.36)	(0.35)	(0.35)	(0.29)	(0.021)	(0.030)
Other vegetables	0.45	0.46	0.45	0.80^b^	0.78	0.75^b^	0.042	0.023
	(0.50)	(0.50)	(0.50)	(0.40)	(0.41)	(0.43)	(0.030)	(0.035)
Other fruits	0.08^a^	0.12^a^	0.09	0.12	0.10^c^	0.13^c^	−0.018	0.064***
	(0.27)	(0.32)	(0.29)	(0.33)	(0.30)	(0.33)	(0.020)	(0.022)
N	733	771	985	733	771	985	2258	1363

Source: Authors' calculations.

Notes: Standard deviations in parentheses. NI: Nutrition Intensification. Superscripts a, b and c denote significant differences (p < 0.1) between NI and Standard arms, NI and Comparison arms, and Standard and Comparison arms, respectively. *** p < 0.01; ** p < 0.05; * p < 0.10. Column (7) reports the DID estimates for PRADAN (NI+Standard) versus the Comparison arm. The ‘normal’ range for BMI is 18.5–24.9 kg/m^2^; BMIs above and below this range are classified as overweight, and underweight, respectively.

For the treatment and comparison samples matched using baseline characteristics, we estimate the following for individual i in household h in district d in state s and for those outcomes available at both baseline and endline:

(1) $$\Delta Y_{\mathrm{ihds}}=\alpha +\beta Treat_{\mathrm{ihds}}+{∊}_{\mathrm{ihds}}$$

Where $\Delta Y_{\mathrm{ihds}}=Y_{\mathrm{ihds}t_{1}}-Y_{\mathrm{ihds}t_{0}}$, i.e., the difference in the outcome between baseline ($t_{0})$ and endline ($t_{1})$; and $\mathrm{Trea}t_{\mathrm{ihds}}$ is an indicator for individual i’s treatment status. For the comparison between PRADAN and non-PRADAN, this is 1 if the individual belongs to either the NI or Standard arms. For the comparison between NI and Standard arms, this is 1 if the individual belongs to the NI arm.

For those outcomes measured only at endline, we replace $\Delta Y_{\mathrm{ihds}}$ in equation (1) with $Y_{\mathrm{ihds}t_{1}}$, and estimate this modified equation on the matched treatment and comparison samples as above.^4^

Evaluating a large, complex intervention implemented by an organization that has been working in communities for an extended period poses challenges. The empirical strategy outlined above was the best available to us but does have two key limitations. First, since we do not have data on these households prior to the baseline, we are unable to test for pre-trends in our key outcomes of interest. While the study design appears to have achieved balance in characteristics (Table 1), we do see statistically significant differences in our key outcomes at baseline (see Section 4 below). Second, we were not able to randomize the rollout of the treatment, as the intervention areas were pre-selected by the implementer. The block- and individual-level matching attempts to account for placement and selection biases but cannot fully address this issue. Thus, in the rest of the paper we acknowledge that our results do not provide causal estimates.

### 3.4 Outcomes

Our primary outcomes are the respondent woman’s BMI and dietary diversity. Power calculations conducted prior to baseline using secondary data from Madhya Pradesh and Odisha showed that a total sample size of 800 women in 8 block-level clusters would give us 80 % power to detect a difference of 0.4–0.6 in women’s BMI and a difference of 0.17 in women’s dietary diversity score.

In addition to BMI, we look at changes in indicators for the respondent woman being underweight, of normal weight and overweight.^5^ We measure dietary diversity using (1) the number of distinct food groups consumed in the previous 24 h; and (2) whether the woman achieves minimum dietary diversity, defined as consuming at least 5 out of 10 food groups (WDDP Study Group, 2017). We also investigate changes in the consumption of individual food groups that contribute towards dietary diversity. We hypothesize that both dietary diversity and BMI would be higher in the NI relative to the Standard arm; this is a low-BMI population where increases in BMI would not be associated with a higher prevalence of obesity.

We then examine intermediate outcomes along the income, agriculture, health and nutrition behavior change, and rights impact pathways and the women’s empowerment cross-cutting pathway.

*Behavior change pathway:* Women’s knowledge of the BCC content, including knowledge scores on breastfeeding and IYCF, complementary feeding and IYCF at different ages of the child (6 months, 6–8 months), nutrition of pregnant women and anemia, and child health, hygiene and sanitation. In addition, outcome variables include their exposure to the BCC content, measured by their recall of the key characters in the stories included in the BCC modules (women named Soni, Madhu and Silvanti), their recall of any stories containing messages on key topics such as early marriage, registering in the Anganwadi center during pregnancy, taking care of newborn babies and so on, and their participation in community health and nutrition events such as BMI or haemoglobin camps.

We hypothesize that participants in the NI arm would have higher knowledge scores, exposure, recall, and participation in community health and nutrition events relative to those in the Standard arm.

*Income pathway:* Per capita monthly expenditures (total, food, and non-food); per capita expenditures on animal source foods, and the first principal component on a list of assets (hereafter called Wealth PCA). Relative to the Comparison group, we hypothesize that both Standard and NI arms would have higher per capita expenditures and wealth, but that there would be no difference between NI and Standard arms given that there were no resource transfers as part of the NI intervention.

*Agriculture pathway:* Indicators related to home garden cultivation. We hypothesize that the participants in the NI arm would be more engaged in home garden cultivation relative to those in the Standard arm.

*Rights pathway:* Government schemes awareness and benefits scores. We hypothesize that both the Standard and NI arms would have higher scores than the Comparison arm, and that women in the NI arm would have higher scores related to the utilization of health and nutrition services than those in the Standard arm.

*Women’s empowerment pathway:* Women’s and men’s empowerment scores and the intrahousehold inequality score, using the A-WEAI. Since the nutrition intensification intervention did not have a women’s empowerment component, we do not expect impacts on empowerment scores or intrahousehold inequality in the NI relative to the Standard arm. However, we expect that PRADAN members would have higher empowerment scores given the women-focused nature of SHG programming.

## 4 Results

### 4.1 Primary outcomes: women’s BMI and diets

At baseline, average BMI among non-pregnant women was 19.23 (NI), 19.28 (Standard) and 18.92 (Comparison) kg/m^2^ (Table 2) using typical BMI cutoffs, and 45–48 percent of the women were underweight. Diets were poor, with women consuming an average of 2.84 food groups in the NI arm in the previous 24 h, 2.98 food groups in the Standard arm and 2.83 food groups in the Comparison arm, out of a possible ten (Table 2). Only 9 percent of women in the NI arm, 13 percent in the Standard arm and 8 percent in the Comparison arm attained minimum dietary diversity (defined as consuming at least 5 out of the 10 food groups in a 24-hour period). Between 17 and 23 percent of women reported consuming animal sourced foods (eggs, meat, chicken, fish) in the preceding 24 h. Almost all women consumed starchy staples at baseline and close to half consumed pulses, dark green leafy vegetables, and other vegetables. Consumption of dairy, eggs, flesh foods, nuts and seeds and vitamin A-rich fruits and vegetables was low, with fewer than a fifth of women consuming these food groups.

Low baseline levels of consumption of nutrient-rich foods indicate considerable scope for improvement in diet quality. However, while we observe an overall secular improvement in BMI and dietary diversity between baseline and endline, we find do not find significant associations between being in the PRADAN intervention or the NI arm on BMI and dietary diversity. There is a small positive increase observed in either the Standard or NI arm on the consumption of starchy staples and nuts and seeds, and a counterintuitive decrease in the consumption of pulses. Nevertheless, pulses consumption improves in all arms, especially in the Standard arm.

Comparing women in the NI and Standard arms, we observe increases in the consumption of nuts and seeds, flesh foods, animal source foods, and other fruits, possibly owing to BCC around diet quality and the importance of eating nutrient-rich foods in the NI arm.

The improvements in intake of nutritious foods in the NI arm are consistent with findings from the process evaluation. Dietary diversity and healthy diets were the explicit focus of several modules and mentioned in later modules; while most topics lasted for one month, activities directly related to diet lasted for 2–3 months (Nichols, 2021b). Messaging around dietary diversity also had simple, catchy phrases, like *‘teen rang ka thali’* (three-color plate, denoting a plate with starch, vegetable, and protein). Moreover, women viewed the recommendation to eat diverse diets as socially desirable. Before the intervention, many women did not prioritize dietary diversity, which seemed like a luxury. However, the messaging in the intervention emphasized immediate benefits such as improved energy and strength (to secure livelihoods) and boosted immune systems (to reduce healthcare costs).

### 4.2 Outcomes along the impact pathways

#### 4.2.1 Health and nutrition behavior change pathway

At baseline, the average breastfeeding knowledge score was 42.6 (NI), 39.6 (Standard) and 38.8 (Comparison), out of a maximum of 100 (Table 3). Knowledge scores on complementary feeding practices were even lower on average – with scores ranging from 25 to 29 out of 100. Scores for knowledge of child health, hygiene and sanitation were 54.9 (NI), 57.1 (Standard) and 53.3 (Comparison).

**Table 3:** Trends and DID estimates of impacts on knowledge scores.

	**Baseline Means**			**Endline Means**			**DID**	
	**NI**	**Standard**	**Comparison**	**NI**	**Standard**	**Comparison**	**PRADAN vs Comparison**	**NI vs Standard**
	(1)	(2)	(3)	(4)	(5)	(6)	(7)	(8)
Breastfeeding, IYCF	42.58^a|b^	39.64^a^	38.81^b^	52.90^a|b^	49.13^a^	50.20^b^	−0.898	0.419
	(19.37)	(19.13)	(18.10)	(18.16)	(18.95)	(18.77)	(1.157)	(1.452)
Complementary feeding, IYCF (at 6 months)	25.17^b^	25.68^c^	29.34^b|c^	16.77^b^	16.23^c^	14.25^b|c^	4.165***	1.325
	(26.75)	(27.81)	(28.04)	(22.80)	(21.34)	(22.08)	(1.579)	(1.982)
Nutrition for pregnant woman and anemia	48.28	48.96	46.80	81.72	80.23	81.57	−2.395	3.074
	(34.57)	(33.45)	(34.67)	(22.28)	(24.24)	(23.05)	(1.716)	(2.068)
Child health, hygiene and sanitation	54.98	57.07^c^	53.33^c^	66.34	63.89	64.05	0.235	4.827**
	(33.35)	(32.95)	(32.97)	(30.74)	(31.03)	(30.68)	(1.930)	(2.254)
N	733	771	985	733	771	985	2460	1478

Source: Authors' calculations.

Notes: Standard deviations in parentheses. NI: Nutrition Intensification. Superscripts a, b and c denote significant differences (p < 0.1) between NI and Standard arms, NI and Comparison arms, and Standard and Comparison arms, respectively. NI: Nutrition Intensification. *** p < 0.01; ** p < 0.05; * p < 0.10. Column (7) reports the DID estimates for PRADAN (NI+Standard) versus the Comparison arm.

Knowledge scores on all topics except complementary feeding increased over time across all arms (Table 3); knowledge scores for complementary feeding decreased in all arms. We do find an increase in knowledge of complementary feeding among women in PRADAN areas compared to those in Comparison areas, and on knowledge of child health, hygiene, and sanitation for women in the NI arm relative to the Standard arm. We did not find any other significant differences.

Changes in outcomes along the health and nutrition pathway are closely linked to exposure to the intervention. Table 4 captures exposure to health and nutrition intervention components at endline, including the respondents’ recognition of key characters in the BCC material and key messages in the BCC stories, and having participated in community events. Exposure to key intervention components was extremely low in the NI arm at endline, suggesting diluted implementation intensity; only 15–21 % of respondents in the NI arm had heard of key characters and very few (4–16 %) reported having attended any community events. Given such low exposure, the modest changes in knowledge are unsurprising.

**Table 4:** Exposure to the health and nutrition intervention components at endline.

	**Endline means**		
	**NI**	**Standard**	**Comparison**
**PANEL A: Whether respondent woman has heard of:**			
Soni’s story	0.21^a|b^	0.02^a^	0.01^b^
	(0.41)	(0.15)	(0.11
Madhu’s story	0.19^a|b^	0.02^a|c^	0.01^b|c^
	(0.39)	(0.14)	(0.11)
Silvanti’s dream	0.15^a|b^	0.02^a^	0.01^b^
	(0.35)	(0.13)	(0.09)
**PANEL B: Whether respondent woman has heard any story about:**			
Early marriage or early pregnancy	0.25^a|b^	0.06^a|c^	0.02^b|c^
	(0.44)	(0.24)	(0.13)
Registering in Anganwadi center during pregnancy	0.22^a|b^	0.06^a|c^	0.02^b|c^
	(0.41)	(0.23)	(0.14)
Different foods to eat and grow in home gardens	0.22^a|b^	0.05^a|c^	0.02^b|c^
	(0.41)	(0.22)	(0.12)
A woman who has anemia and gets sick	0.19^a|b^	0.04^a|c^	0.02^b|c^
	(0.39)	(0.20)	(0.13)
Delivery and small families	0.17^a|b^	0.03^a|c^	0.01^b|c^
	(0.37)	(0.18)	(0.10)
Taking care of newborn babies	0.17^a|b^	0.05^a|c^	0.01^b|c^
	(0.38)	(0.21)	(0.11)
Taking care of children > 6 months	0.14^a|b^	0.04^a|c^	0.01^b|c^
	(0.35)	(0.20)	(0.11)
Voting and government entitlements	0.16^a|b^	0.07^a|c^	0.01^b|c^
	(0.36)	(0.26)	(0.10)
**PANEL C: Participation in community events**			
BMI camps	0.16^a|b^	0.06^a|c^	0.03^b|c^
	(0.36)	(0.23)	(0.16)
Haemoglobin camps	0.10^b^	0.08^c^	0.03^b|c^
	(0.30)	(0.27)	(0.18)
Health and nutrition fair	0.05^a|b^	0.02^a|c^	0.01^b|c^
	(0.22)	(0.14)	(0.07)
Nutritious food fair in village	0.05^a|b^	0.02^a|c^	0.00^b|c^
	(0.22)	(0.13)	(0.03)
Village-level nutrition and health meeting	0.08^a|b^	0.03^a^	0.02^b^
	(0.26)	(0.17)	(0.15)
Large health meeting	0.04^a|b^	0.01^a|c^	0.00^b|c^
	(0.19)	(0.12)	(0.06)
*N*	733	771	985

Source: Authors' calculations.

Notes: Standard deviations in parentheses. NI: Nutrition Intensification. Superscripts a, b and c denote significant differences (p < 0.1) between NI and Standard arms, NI and Comparison arms, and Standard and Comparison arms, respectively. Soni, Madhu and Silvanti are key female characters in the stories included in the BCC content.

These results are confirmed by the process evaluation (Nichols, 2021b). The process evaluation showed that low exposure to messages, uneven dissemination of nutrition information, and content and delivery of information were barriers to impact along the BCC pathway. Although SHGs in our study areas were largely functional and performed the expected savings and credit activities, the delivery of nutrition BCC messages by PSs and other program staff to SHG members was not as widespread or successful. At endline, the reported name recognition of key characters in the stories used to deliver messages was low in all arms (Table 4) though considerably higher in the NI arm compared to Standard and Comparison arms. Exposure rates based on topical recall were also low, but a large fraction of those who had heard the message reported hearing it at an SHG meeting rather than from any other program.

Another barrier along the BCC pathway was the limited time, incentive structure, and training of PSs, who were the heart of the intervention. Only 56% of the PSs reported receiving any health and nutrition training as part of the intervention and only 65% reported discussing health and nutrition topics at SHG meetings. PSs were required to travel to SHGs’ locations to conduct the BCC sessions, a significant time commitment especially during busy agricultural months, and were often unable to meet all the SHGs within their purview each month. Refresher trainings held in block towns or nearby villages were also crucial, yet PSs also faced challenges to attending regularly owing to time and travel constraints. Similarly, PSs would face difficulties when SHGs did not meet or members did not stay for the entire duration of the meeting (Nichols, 2024).

The high volume of messages delivered led to low retention, even among the PSs, especially for messages delivered earlier in the BCC cycle. The mode of delivery may also have played a role, with better recollection of picture cards than the specific details of each story. Nor was the content of the stories well targeted to the SHG audience. Respondent women were 33 years old on average and only 12 percent had children under 2 years at baseline, therefore complementary feeding was not relevant at baseline or endline, possibly contributing to the decline in knowledge about these practices over time. The intervention’s strong focus on IYCF practices had little relevance for older SHG members who were past their peak childbearing years, while only a few messages in the BCC curriculum were targeted towards women’s own nutrition and health and were often framed in terms of their implications on children. However, promoting healthy diets is an appropriate intervention regardless of lifecycle stage, and healthy, diverse diets for women themselves were actively promoted.

Stories around early marriage/childbirth and dietary diversity were more easily recalled because these resonated with a broader set of women, were woven throughout the curriculum, and despite delving into complex topics, were shorter, simpler stories with pithy catchphrases (*kam umr ki shaadi* (“marriage at a young age”) *and teen rang ki thali* (“tri-coloured plate”)) (Nichols, 2021b). Exposure to these messages, while higher than others, was still low, indicative of additional barriers to messages reaching women.

Much of the PRADAN model relied on diffusion of information by SHG members. While we did not find evidence of active diffusion, it is true that even if older women—mothers and mothers-in-law—do not themselves have young children, in the South Asian context, they are important decisionmakers within the household and are considered influencers for younger women of the family.

#### 4.2.2 Income pathway

Because livelihoods-based activities for income generation did not differ across the Standard and the NI arms, we do not anticipate that household total incomes will differ across treatment arms in PRADAN areas. Somewhat surprisingly, we observe increases in per capita total expenditure and per capita food expenditure in the NI relative to the Standard arm, but no significant differences in per capita expenditure in the PRADAN areas compared to the Comparison areas, except for expenditures on animal source foods (Table 5, Panel A).

**Table 5:** Impact on intermediate outcomes along the impact pathways.

	**DID impact estimate** **PRADAN vs Comparison**	**Outcome indicator endline mean** **Comparison arm**	**DID impact estimate** **NI vs Standard**	**Outcome indicator endline mean,** **Standard arm**
	(1)	(2)	(3)	(4)
**Panel A: Income pathway** *(DID with nearest neighbor matching)*				
Total per capita monthly expenditure	86.423	*932.05*	150.606**	*966.33*
	(61.271)		(69.550)	
Per capita monthly non-food expenditure	52.528	*485.48*	32.329	*548.62*
*(N column 1: 2455, N column 3: 1475)*	(55.226)		(63.114)	
Per capita monthly food expenditure	34.786	*447.56*	120.061***	*420.23*
*(N column 1: 2457, N column 3: 1475)*	(23.983)		(26.807)	
Per capita monthly animal-sourced food expenditure	21.296***	*22.76*	9.041	*22.15*
*(N column 1: 2457, N column 3: 1475)*	(8.218)		(6.318)	
Wealth PCA	0.164***	*−0.09*	0.018	*0.10*
*(N column 1: 2377, N column 3: 1432)*	(0.063)		(0.087)	
**Panel B: Agriculture pathway** *(Single difference with nearest neighbor matching)*				
Ever heard of home gardens	0.043***	*0.880*	0.039**	*0.885*
	(0.014)		(0.017)	
Currently have a home garden	0.032	*0.425*	0.091***	*0.410*
	(0.022)		(0.029)	
Home garden cultivation:				
No. of vegetables cultivated	0.236*	*1.967*	0.704***	*1.824*
	(0.128)		(0.161)	
No. of fruits cultivated	0.025	*0.649*	0.114	*0.586*
	(0.055)		(0.072)	
Grow any vit A rich fruits or vegetables	0.032	*0.270*	0.061**	*0.258*
	(0.020)		(0.026)	
Grow any dark green leafy veg	0.015	*0.065*	0.041***	*0.043*
	(0.011)		(0.014)	
Grow plants year-round	0.062***	*0.071*	0.100***	*0.079*
	(0.013)		(0.020)	
**Panel C: Rights pathway** *(DID with nearest neighbor matching)*				
Government schemes:				
Awareness score (0–100)	−0.216	*92.179*	1.266	*91.745*
	(0.584)		(0.788)	
Benefits score (0–100)	1.929*	*51.731*	0.461	*55.111*
	(1.018)		(1.315)	
**Panel D: Women’s empowerment pathway** *(DID, IPW regressions with selection bias weights)*				
**Abbreviated-WEAI composite indicators**				
Women’s empowerment score	0.006	*0.550*	0.014	*0.570*
*(N column 1: 1333, N column 3: 788)*	(0.018)		(0.021)	
Men’s empowerment score	−0.038**	*0.693*	−0.021	*0.685*
*(N column 1: 1330, N column 3: 786)*	(0.016)		(0.022)	
Intrahousehold inequality score	−0.017	*0.119*	−0.017	*0.095*
*(N column 1: 1330, N column 3: 786)*	(0.015)		(0.017)	

Source: Authors’ calculations. Notes: Number of observations is 2460 and1478 in columns 1 and 3, respectively, unless otherwise mentioned. Standard errors in parentheses. NI: Nutrition Intensification. *** p < 0.01; ** p < 0.05; * p < 0.10. Column (1) reports the DID estimates for PRADAN (NI+Standard) versus the Comparison arm. All variables in monetary units were deflated using the Consumer Price Index in 2015 for the general rural population.

Note that because the intervention did not deliver cash or in-kind transfers along with the BCC, resource, time, and agency constraints may have limited women’s ability to implement recommended behaviors or even attend meetings to learn about interventions they could not afford to implement (Nichols, 2021a, Nichols, 2021b). We find that women were more likely to adopt low-cost WASH-related behaviors, such as handwashing, than more costly diet-related ones, such as eating more animal-sourced foods or dark green leafy vegetables. Our qualitative work indicates that the resource constraint may have been more binding than deeply engrained cultural/religious dietary habits (Nichols, 2021b).

#### 4.2.3 Agriculture pathway

We do expect differential improvements in some agricultural outcomes between NI and Standard arms, especially increased cultivation of home gardens and specific varieties of crops cultivated (Table 5, Panel B). We observe an increase in awareness of home gardens, number of vegetables cultivated, and the probability of cultivating a home garden year-round in households in PRADAN relative to Comparison areas. Households in the NI arm are more likely than those in the Standard arm to have heard of home gardens, to currently cultivate a home garden, to cultivate a home garden year-round, and to grow a larger number of vitamin A rich fruit and vegetables and dark green leafy vegetables. These estimated improvements in home garden related outcomes are based on single-difference estimates using endline data only.

According to the process evaluation (Nichols, 2021b), immediately after the dietary diversity module introduced the benefits from different food groups, a kitchen garden module was introduced, addressing food access issues and reinforcing the importance of dietary diversity. In one site, where chemical agriculture was widespread, SHGs members enjoyed eating organic vegetables from home gardens, and felt it was better to grow vegetables without chemicals than purchase them from the market. The interlinked modules on dietary diversity and kitchen gardens “validated and encouraged women’s existing practices while celebrating their cultural heritage and embodied knowledge around food.” (Nichols, 2021b p. 8).

#### 4.2.4 Rights pathway

Since awareness of government schemes was almost universal (>91 percent) in the Comparison arm at endline, it is unsurprising that respondents in PRADAN areas did not demonstrate significantly greater awareness of such schemes. Households in the PRADAN areas received marginally greater benefits from the government schemes than Comparison group households (Table 5, Panel C); however, this difference is quite small, only 1.9 percent more compared to the Comparison group endline mean of 51.7 percent. Excluding government schemes that are targeted to pregnant women does not change our results significantly.

Findings from the process evaluation are insightful. (Nichols, 2021b) found that although the curriculum was designed to be delivered through a PLA approach, its implementation was like more conventional didactic BCC approaches. As a result, some of the more transformational aims of PLA, such as mobilizing communities to demand government services, were not achieved.

#### 4.2.5 Women’s empowerment pathway

The subsample for the women’s empowerment analysis, which was restricted to households with both a male and a female respondent, was systematically different from the overall sample. We estimate an inverse probability weighted regression model with weights defined as the product of the sample selection weight and the propensity score (estimated from the matching models) to account for potential bias. The NI intervention affected neither the probability that the woman was empowered nor the women’s empowerment score, but the men’s empowerment score in households in PRADAN decreased slightly relative to Comparison areas (Table 5, Panel D). The null findings for empowerment indicators is expected given the absence of specific women’s empowerment-related activities in addition to standard PRADAN programming. Although we have documented significant positive associations of SHG membership with women’s empowerment (Kumar et al., 2021)*,* the NI interventions did not confer additional empowerment benefits.

The null results on women’s empowerment indicators, which are indicative of women’s ability to make strategic decisions and act on them, suggest that the intervention did not do much to change gender norms or women’s decisionmaking power within the household. Thus, women may have felt they did not have agency to implement what they learned.

Group membership challenges may also have limited women’s ability to build collective agency, an aspect of empowerment. Creating an effective SHG platform for service delivery requires substantial time investment and skill by its members, which is driven in part by the perceived value of group participation and trust in other group members. The process evaluation (Nichols, 2021a) suggests that women who had not yet received benefits from SHG membership (e.g., loans, training, confidence) were less interested in participating in nutrition meetings than women who had received benefits and developed greater trust in the collective as an institution.

## 5 Discussion

We assessed the effectiveness of a health and nutrition BCC intervention layered on to an agricultural livelihoods-based women’s SHG platform and found that our primary outcomes of women’s BMI or overall dietary diversity did not improve in the NI arm relative to the Standard and Comparison arms. While women increased consumption of animal source foods, nuts and seeds, and fruits in the NI arm, this was not enough to increase overall dietary diversity scores or the proportion achieving minimum dietary diversity. Key intermediate outcomes along several hypothesized impact pathways improved, such as increased adoption of home gardens, but not on the health and nutrition BCC pathway, central to the intervention, nor the women’s empowerment pathway. Results from qualitative studies undertaken as part of a mid-term process evaluation provided insights into enablers and barriers along the hypothesized impact pathways.

### 5.1 Enablers and barriers

Desai et al. (2021)’s systematic review discusses the roles that context, intervention design and implementation, and outcome characteristics play in relation to the success (or lack thereof) of women’s group-based interventions to improve health and nutrition outcomes in India. Viewed in that light, the findings from this study are not unusual. Among the contextual barriers to success were lack of adequate health and nutrition services in rural areas. Although the NI intervention addressed some barriers to better maternal nutrition such as income and access to nutritious foods through home garden cultivation, many of the behaviors recommended by the BCC curriculum depended on the availability of services.

Comparisons with other trials conducted in India are instructive. The CARING trial, implemented in two states in India, strengthened service delivery by introducing a new cadre of community-based workers to improve nutrition (Nair et al., 2017). Groups of young women were formed specifically for BCC, but others beyond the core members were also invited to join. Compared to the PRADAN NI interventions, these interventions were more intensive—they included follow-up home visits for targeted beneficiaries and relied on paid, not volunteer, health workers. Despite significant improvements in diets, handwashing, infant survival and a decline in the probability of being underweight at 18 months, the intervention had no impact on linear growth. Another intervention, Action Against Malnutrition (AAM), was a civil society-led community-based initiative to supplement the efforts of frontline health and nutrition workers in four states in India (Gope et al., 2019). The intervention included monthly participatory learning and action (PLA) meetings with women’s groups followed by counselling through home visits in some areas and crèches for children aged 6 months to 3 years plus the PLA meetings and home visits in others. The crèches provided an opportunity to co-locate free care in a safe, smoke-free environment with clean drinking water, handwashing stations, nutritious food, growth monitoring, and psychosocial stimulation, thus delivering a more intensive treatment. The impact evaluation of AAM found significant reduction in wasting, underweight and stunting among children under 3 years old. However, while infant and young child feeding practices improved in both home visit and creche areas, consumption of protein-rich foods improved and stunting declined only for those households with access to the creches.

Intervention design and implementation also played a role in the lukewarm performance of the NI intervention. Desai et al. (2021) point out that groups that improved health outcomes did not aim to “nudge” new behaviors but built individual, group, and communities’ capabilities by encouraging participation, problem solving, and locally relevant solutions to address direct and underlying determinants of health and nutrition behavior. Key to the success of these programs were motivated and well-trained facilitators, often community women. However, our process evaluation shows that even among the PSs who were trained specifically to deliver nutrition messages, retention of messages was low (Nichols, 2021b). Moreover, the effective group interventions identified by the review attained sufficient intervention intensity with meetings held at least monthly and over a year or more. In this NI intervention, the nutrition information was given in the second part of the meeting, and SHG members, particularly those with time and resource constraints, did not stay to participate in the discussion of nutrition messages. Because this was a program without any resource transfers, material and time constraints faced by SHG members were not alleviated. Indeed, Keats et al. (2021) found that in food insecure settings, positive impacts on nutrition were observed only when nutrition education programs were combined with supplementary food transfers.

Desai et al. (2021) also argue that relevance and salience of topics discussed affected the degree to which women and community members participated in group activities. In the NI intervention, discussions of ICYF may not have reached their intended audience if SHG members tended to be older, even if mothers-in-law are important influencers within the household.

Finally, the type of women’s group may matter, with “club” approaches that invested in group strength and actively facilitating group action for health reporting positive impacts, and collectives that invested time in participatory approaches reporting more improvements in outcomes at a population level (Desai et al. 2021: 9). Although the NI intervention attempted to use some elements of the PLA approach, inexperienced facilitators ended up reverting to a more didactic model that did not necessarily engage participants in participatory learning.

### 5.2 Other concurrent nutrition-focused programs

Table 2, Table 3 show that women in the Comparison arm were doing as well (or in some cases, better) than one or more of the two treatment arms on key nutrition indicators relating to anthropometry and diet. In several instances, the Comparison arm women demonstrated more rapid change in these indicators between baseline and endline than women in the treatment arms, for example, in the case of BMI, prevalence of underweight and average weight, total number of food groups consumed in the preceding 24 h, and several knowledge indicators, such as on breastfeeding and pregnant women’s anemia and nutrition.

This seemingly curious finding can be explained in the context of the large national nutrition-focused program – the National Nutrition Mission (NNM) − that was implemented concurrent to this evaluation and would have served to improve outcomes across the board. This program was announced in 2017 as a flagship government program and launched in early 2018, and by 2019, around the time of the endline for this evaluation, the implementation of the NNM had begun in all districts of the country. Key NNM activities were very similar to the NI intervention we evaluate in this paper. In particular, there was a strong focus on a behavior change campaign that leveraged multiple platforms and touched upon many of the topics included in the NI BCC materials such as breastfeeding and complementary feeding, anemia prevention, early marriage and so on. The two-year overlap between the PRADAN NI intervention and the NNM program could explain the impressive improvements in health and nutrition indicators in the Comparison arm. More details can be found in (Scott et al., 2022).

## 6 Conclusions and policy implications

The ability of SHG platforms to successfully deliver behavior change interventions to improve women’s diets and nutrition depends on a range of factors: whether the groups exist, are functional, and meet for a sufficient duration; whether recipients can and do process the information provided, have the resources and agency to apply it in their daily lives, and share it with others; and whether different agents tasked with information dissemination have the resources and support to execute their roles effectively. Based on both a quantitative impact evaluation and a mixed methods process evaluation, our findings suggest the need for more realistic expectations of the ability of BCC programs to improve nutrition specifically, and to scale up service delivery more generally. This is particularly true when the intervention is being evaluated only after a few years of implementation, which may not be long enough to achieve uptake, much less affect behavioral outcomes or social norms.

Despite the absence of data to test the parallel trends assumption, the consistent findings from the quantitative impact evaluation and the mixed methods process evaluation allow us to draw out key policy implications. Among these is the need to temper the enthusiasm for using women’s group platforms for service delivery. Overloading existing platforms to deliver even more services without devoting additional resources either to beneficiaries or as incentives to frontline agents will most likely be counterproductive.

Retrofitting an existing platform to provide additional services may not be straightforward; it may require additional personnel and monitoring mechanisms, and depending on the specific service, may either not address or (at worst) exacerbate existing inequities. In the absence of appropriate investments in human resources, many group-based programs end up reaching the same people, usually richer or better-connected women, despite trying to reach those who are marginalized and resource- or time-poor. Moreover, without providing additional in-kind benefits or creating policies that equalize gendered labor inequities, providing BCC to marginalized or resource poor women may not necessarily be empowering.

Further efforts to use group-based platforms as avenues for service delivery would do well to strengthen the collective agency built by those groups while being mindful of the time and resource constraints that individual women face.

## CRediT authorship contribution statement

**Neha Kumar:** Writing – review & editing, Writing – original draft, Validation, Supervision, Resources, Project administration, Methodology, Investigation, Funding acquisition, Formal analysis, Data curation, Conceptualization. **Kalyani Raghunathan:** Writing – review & editing, Writing – original draft, Methodology, Investigation, Formal analysis, Data curation. **Agnes Quisumbing:** Writing – review & editing, Writing – original draft, Methodology, Investigation, Formal analysis, Conceptualization. **Samuel Scott:** Writing – review & editing, Methodology, Investigation, Formal analysis, Conceptualization. **Purnima Menon:** Writing – review & editing, Methodology, Investigation, Funding acquisition, Conceptualization. **Giang Thai:** Writing – review & editing, Formal analysis, Data curation. **Shivani Gupta:** Writing – review & editing, Formal analysis, Data curation. **Carly Nichols:** Writing – review & editing, Writing – original draft.

## Declaration of Competing Interest

The authors declare that they have no known competing financial interests or personal relationships that could have appeared to influence the work reported in this paper.

## Footnotes

2 In the Indian context, SHGs are women’s SHGs.

3 See https://nrlm.gov.in/outerReportAction.do?methodName=showIndex#gsc.tab=0.

4 Equation (1) shows the estimation model as a linear model. However, as mentioned in this section, we estimate treatment effects using nearest neighbor matching, which is nonlinear.

5 Body mass index (BMI) is calculated as the ratio of weight (in kilograms (kg)) to height-squared (in meters-squared). The ‘normal’ range for this indicator is 18.5–24.9 kg/m2. BMIs above and below this range are classified as overweight and underweight, respectively.
